# A High-Throughput Assay for Monitoring Ubiquitination in Real Time

**DOI:** 10.3389/fchem.2019.00816

**Published:** 2019-12-04

**Authors:** Tyler G. Franklin, Jonathan N. Pruneda

**Affiliations:** Department of Molecular Microbiology and Immunology, Oregon Health & Science University, Portland, OR, United States

**Keywords:** ubiquitin, high-throughput screen, fluorescence polarization, ubiquitin ligase, deubiquitinase

## Abstract

Protein ubiquitination is a highly orchestrated process that controls diverse aspects of human biology. Dysregulation of this process can lead to various disease states including cancer, neurodegeneration, and autoimmunity. It is the correction of these dysregulated pathways, as well as the enticing ability to manipulate protein stability, that have instigated intense research into the therapeutic control of protein ubiquitination. A major bottleneck in the development and validation of small molecule modulators is the availability of a suitable high-throughput assay for enzyme activity. Herein, we present a new assay, which we term UbiReal, that uses fluorescence polarization to monitor all stages of Ub conjugation and deconjugation in real time. We use the assay to validate a chemical inhibitor of the E1 ubiquitin-activating enzyme, as well as to assess the activities and specificities of E2s, E3s, and deubiquitinases. The sensitivity and accessibility of this approach make it an excellent candidate for high-throughput screens of activity modulators, as well as a valuable tool for basic research into the mechanisms of ubiquitin regulation.

## Introduction

Post-translational regulation through attachment of the small protein modifier ubiquitin (Ub) is a conserved and essential process among all eukaryotic life. Protein ubiquitination can regulate diverse cellular processes including proteasomal degradation as well as protein trafficking, cell cycle regulation, and immune signaling (Komander and Rape, 2012). Ub is typically attached via its carboxy-terminus to a lysine residue on a target protein, resulting in a monoUb modification. The vast diversity of Ub signaling roles arises from additional customization of the monoUb signal. Unlike binary post-translational modifications such as phosphorylation or acetylation, Ub itself is a protein and can thus be further post-translationally modified by e.g., ubiquitination. Ubiquitination of Ub can occur at any of eight classical sites (seven lysine positions and the amino-terminus), creating an array of polymeric Ub (polyUb) chains. MonoUb as well as each polyUb chain type are believed to serve distinct signaling roles, for example chains linked through K48 are the classic proteasomal degradation signal, whereas Met1-linked polyUb serves a specialized role in innate immune signaling (Komander and Rape, 2012; Swatek and Komander, 2016). Additionally, target proteins can be ubiquitinated at multiple sites, further diversifying the versatility of Ub signaling.

**Figure 1:**
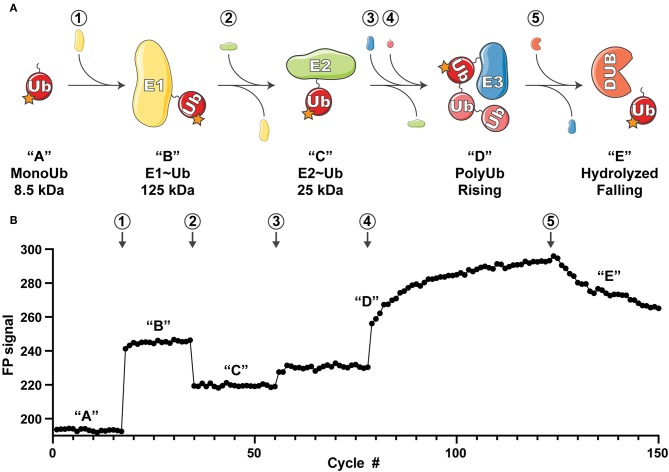
Overview of the UbiReal approach. **(A)** Schematic depicting the sequential states of ubiquitin conjugation that are monitored using UbiReal. Circled numbers indicate the addition of protein and correspond to the data in part **(B)**. Letters in quotations indicate the state of the ubiquitin complex and also correspond to the changes in FP signal observed in (B). Approximate molecular weights of the complexes are provided and reflect the amplitude of the expected FP signal. “Rising” in “D” corresponds to the rising FP signal from the ligation of chains by the E3 enzyme, and “falling” in “E” corresponds to falling FP signal from the activity of a DUB enzyme. (B) Data representing the ability of UbiReal to comprehensively monitor the sequential steps of the ubiquitination pathway. Numbers and letters correspond to protein additions and the states of ubiquitin complexes, respectively, as described in (A). For clarity, data are presented as cycles separated by 30 s. In this representative assay, “1” is the E1 UBE1, “2” is the E2 UBE2D3, “3” is the E3 NleL, “4” is WT Ub, and “5” is the DUB USP21. Graph is the average of two identical, parallel experiments and representative of multiple other UbiReal experimental curves.

In humans, the Ub system is controlled by hundreds of regulatory proteins (Clague et al., 2015). Ubiquitination occurs via a cascade of Ub “writing” enzymes that include an E1 Ub-activating enzyme, an E2 Ub-conjugating enzyme, and an E3 Ub ligase (Figure 1A). The E1 Ub-activating enzyme (of which there are two in humans) consumes ATP to activate the Ub carboxy-terminus onto an E1 active site cysteine, creating a high-energy thioester linkage (E1~Ub). Next, through a transthiolation reaction the Ub is transferred from the E1 to the active site cysteine of an E2 Ub-conjugating enzyme (of which there are ~35 in humans), forming the E2~Ub conjugate. At this stage, the Ub can either be transferred directly onto a substrate lysine in a reaction catalyzed by E3 ligases of the RING/U-box family (of which there are hundreds in humans), or via one additional thioester intermediate in the cases of the HECT and RBR families of E3 ligases (28 and 14 examples in humans, respectively) which utilize their own active site cysteine to receive and transfer Ub onto a substrate. The resulting Ub signals are discriminately interpreted by Ub binding domains (of which there are >150 in humans) that specifically “read” the modification and direct cellular outcomes. Finally, Ub signals can be “erased” by specialized proteases termed deubiquitinases (DUBs, of which there are ~100 in humans) that can edit or recycle the Ub signal back to its monomeric state (Figure 1A).

In total, approximately 5% of human genes encode regulators of Ub signaling. This significant evolutionary investment is illustrative of the strict regulation maintained over Ub signaling across its broad involvement in cellular processes. Perhaps unsurprisingly, breakdown of this regulation can often lead to disease (Popovic et al., 2014). Defects in Ub signaling are linked to many cancers, as the dysregulation of E3 ligase or DUB activities can directly impact the stabilities of tumor suppressors or oncogene products (Kirkin and Dikic, 2011). Ub proteasome system defects are also linked to neurodegenerative disorders, which arise from an inability to degrade toxic protein aggregates (Zheng et al., 2016). In addition to affecting protein stability, aberrant ubiquitination can result in constitutive activation of signaling pathways such as NF-κB, leading to autoimmune diseases or tumor formation (Hu and Sun, 2016).

The Ub system is a major focus of recent pharmaceutical research as it offers the opportunity to “drug the undruggable,” for example by stabilizing tumor suppressors or inducing the degradation of oncogene products (Huang and Dixit, 2016). The posterchild of successful therapeutics targeting the Ub system is bortezomib (Velcade), which blocks proteasomal degradation of ubiquitinated substrates and is an effective treatment for multiple myeloma (Hideshima et al., 2001). Other efforts have instead targeted the stability of individual proteins. For example, inhibitors of the E3 ligase MDM2 show great promise in preventing p53 ubiquitination, thus rescuing it from degradation (Vassilev et al., 2004). Inhibitors have also been designed to specifically block USP7, a deubiquitinase that would otherwise protect MDM2 from Ub-mediated degradation (Kategaya et al., 2017; Lamberto et al., 2017; Pozhidaeva et al., 2017; Turnbull et al., 2017; Gavory et al., 2018). In an alternative approach, protein-targeting chimeric molecules (PROTACs) can be used to induce the degradation of target proteins by recruiting an E3 Ub ligase (Coleman and Crews, 2018). Thus, we are entering a new era of biomedical research centered around controlling the Ub system as a means to correct disease states.

The development of small molecule modulators of ubiquitination activities hinges upon the availability of robust high-throughput screens (HTS) (Macarrón and Hertzberg, 2009). Currently, screens for DUB activity are much more advanced than those for Ub conjugation. The most widely used substrates for high-throughput DUB assays are Ub-AMC or Ub-Rhodamine, which fluoresce only after cleavage (Dang et al., 1998; Hassiepen et al., 2007). Newer classes of mono- or di-ubiquitin substrates contain a *bona fide* isopeptide linkage and allow for reaction monitoring through either fluorescence polarization (FP) or FRET (Ye et al., 2011; Geurink et al., 2012, 2016; Keusekotten et al., 2013). Still, the available DUB substrates for HTS are very simplified, and do not always accurately reflect the genuine ubiquitinated substrate. In the case of Ub conjugation, screens are much less standardized. It seems that no single method can be applied universally to measure the activities of E1, E2, or E3 enzymes (Sun, 2005; Krist et al., 2016; Foote et al., 2017; Park et al., 2017). Further, most assays require a development step which precludes any kinetic measurement in real time (Sun, 2005; De Cesare et al., 2018). A robust and universal HTS to monitor inhibition or activation along each point in the E1-E2-E3 enzyme cascade would be extremely enabling for both mechanistic studies of Ub transfer as well as small molecule modulator screens.

We present a simple HTS, which we term “UbiReal,” that can track all stages of Ub conjugation and deconjugation in real time. Using fluorescently-labeled Ub, we show that every step of the Ub cascade can be measured by FP in a low volume, high-throughput format. Specifically, we demonstrate the utility of UbiReal for measuring E1 activation, E2~Ub discharge and specificity, E3-dependent Ub chain formation, and DUB-dependent hydrolysis. We highlight the utility of UbiReal for studying small molecule modulators by recapitulating the IC_50_ value of the E1 inhibitor PYR-41 (Yang et al., 2007), as well as for answering basic biochemical questions such as E2-E3 pairing and Ub chain specificity. With minimal adjustment, we are confident that this assay could be applied to any E1/E2/E3/DUB system across both Ub and Ub-like (e.g., NEDD8 or SUMO1/2/3) signaling systems, enabling real time measurement of enzyme activities.

## Methods

### Protein Expression and Purification

Fluorescein-Ub (F-Ub), labeled at all primary amines, was purchased from Boston Biochem (U-590). TAMRA-Ub (T-Ub), labeled only at the amino-terminus, was a kind gift from P. Geurink (Leiden University Medical Centre). Wild-type and mutant Ub proteins were prepared according to Pickart and Raasi (2005) with slight modifications. Briefly, Ub was expressed from the pET-17b vector by autoinduction at 37°C for 48 h. Cells were resuspended in 25 mM Tris (pH 8.0), 200 mM sodium chloride and lysed by sonication. The clarified lysate was acidified with perchloric acid to a final concentration of 0.5% v/v. Some Ub mutants were more sensitive to acid precipitation, and in these cases the acid content was limited to 0.2%. The soluble fraction from the acid precipitation was dialyzed into 50 mM sodium acetate (pH 5.0), loaded onto a HiPrep SP FF 16/10 ion exchange column (GE Life Sciences), and eluted with a linear gradient to 500 mM sodium chloride. Ub-containing fractions were pooled, concentrated using an Amicon centrifugal filter (3K MWCO, EMD Millipore), and further purified with a HiLoad Superdex 75 pg size exclusion column equilibrated in 25 mM sodium phosphate (pH 7.4), 150 mM sodium chloride. Purified Ub fractions were pooled, concentrated, and flash frozen for storage at −80°C.

Human E1 (UBE1) was purified by activation to a GST-Ub column, according to Gladkova et al. (2018). UBE2D3, UBE2L3, UBE2N, and NEDD4L were purified from the pGEX6P-1 vector following overnight induction at 18°C with 0.2 mM IPTG. Cells were resuspended in 25 mM Tris (pH 8.0), 200 mM sodium chloride, 2 mM ß-mercaptoethanol and lysed by sonication. The clarified lysate was applied to glutathione agarose resin (Pierce) and washed according to the manufacturer's recommendations. E2s were eluted from the resin by overnight cleavage with GST-3C protease at 4°C, and the resulting protein was dialyzed into 25 mM sodium phosphate (pH 7.4), 150 mM sodium chloride, 1 mM DTT, flash frozen, and stored at −80°C. NleL was purified according to Hospenthal et al. (2013). E4BU was purified according to Nordquist et al. (2010). USP21 was purified according to Ye et al. (2011) with the SUMO tag left intact. OTUB1^*^ and AMSH^*^ were purified according to Michel et al. (2015). ChlaDUB1 was purified according to Pruneda et al. (2016). All proteins were quantified by absorbance at 280 nm.

### General Assay Parameters

T-Ub assays were monitored using fluorescence polarization (FP) on a BMG LabTech ClarioStar instrument using settings suitable for the TAMRA fluorophore with an excitation wavelength of 540 nm, an LP 566 nm dichroic mirror, and an emission wavelength of 590 nm. F-Ub assays were similarly monitored, with an excitation wavelength of 482 nm, an LP 504 nm dichroic mirror, and an emission wavelength of 530 nm. FP experiments were typically 1–2 h in length and FP values were read every 30–60 s with 20 flashes per sample well, unless otherwise noted. FP experiments were performed using Greiner 384-well small-volume HiBase microplates, with samples in 25 mM sodium phosphate (pH 7.4), 150 mM sodium chloride, 10 mM MgCl_2_ at a final volume of 20 μL unless otherwise noted.

Generally, depending on the assay, a master starting solution was prepared with each component shared by all samples in the assay (e.g., E1, MgCl_2_, T-Ub), and distributed to each sample well. The master solution components were calculated so that desired concentrations would be achieved in a final 20 μL volume and a volume of <20 μL master solution could be added to each well. Then, the experimental components (e.g., inhibitors, E2s, ATP, etc.) or buffer were added to sample wells such that the final desired volume of 20 μL was achieved.

### Complete UbiReal Curve Generation

T-Ub at a final concentration of 100 nM in 25 mM sodium phosphate (pH 7.4), 150 mM sodium chloride, 10 mM MgCl_2_ and 5 mM ATP was monitored for 17 cycles. After cycle 17, E1 was added to a final concentration of 125 nM and monitored. After cycle 34, UBE2D3 was added to a final concentration of 300 nM and monitored. After cycle 57, NleL was next added to a final concentration of 700 nM and monitored. After cycle 78, unlabeled WT Ub was added to a final concentration of 25 μM and monitored. Finally, after cycle 124, USP21 was added to a final concentration of 250 nM and monitored to cycle 150. FP readings were paused prior to the addition of protein, and resumed after protein had been added to the sample wells. The UbiReal curve shown is the average of two identical sample wells and is representative of several experiments.

### E1 Inhibition

0.5 μL of E1 inhibitor PYR-41 (Sigma-Aldrich, N2915) dissolved in DMSO at various dilutions was added to sample wells containing 125 nM E1 and 100 nM T-Ub to final PYR-41 concentrations of 75, 50, 33, 25, 20, 16, 10, 8, 6, 2.5, or 0.5 μM. FP was briefly monitored for 10 cycles before initiating the E1~Ub charging reaction with a 1 μL addition of ATP to a final concentration of 5 mM. FP was continuously monitored for approximately 1 h, at which point it had stabilized. An uninhibited control sample that received 0.5 μL of DMSO instead of PYR-41 was used to determine the maximal E1~Ub charging FP signal.

To determine the inhibition of the E1, the FP values for each PYR-41-treated sample were normalized to its starting FP signal before ATP addition (0% activity), and to the final signal of the uninhibited DMSO control, which served as the maximum FP signal in the assay (100% activity). The initial signal in each sample was determined by averaging the 10 values before ATP addition, and the final signal for each sample was determined by averaging the final 10 values. Each sample was prepared in triplicate, and the experiment was performed separately 3 times.

To construct the IC_50_ curve, the unnormalized FP values were used. The final 10 FP values for each sample were averaged and this was used as the final value to plot against the PYR-41 concentration. This was done for each of the 3 separate experiments as before, giving 3 values at each concentration except the 33 μM PYR-41 sample, which had 2 final values. The non-linear regression calculation in GraphPad Prism was used to fit the curve and calculate the final IC_50_ value.

### E2 Amino Acid Reactivity

Master solutions resulting in final concentrations of 25 mM sodium phosphate (pH 7.4), 150 mM sodium chloride, 100 nM F-Ub, 10 mM MgCl_2_, 125 nM E1 and either 5 mM ATP or no ATP were incubated at RT for 10 min before addition to sample wells. FP was monitored for 5 cycles before addition of either UBE2D3 or UBE2L3 to a final concentration of 300 nM, while a subset of UBE2D3 samples also received an addition of E4BU to a final concentration of 2.5 μM. Samples next received an addition of either no amino acid (buffer alone), lysine, or cysteine to a final concentration of 0, 37.5 mM, or 37.5 mM, respectively. Samples were monitored by FP for approximately 2 h.

### E3 Ligase Assay

A master solution resulting in final concentrations of 25 mM sodium phosphate (pH 7.4), 150 mM sodium chloride, 10 mM MgCl_2_, 100 nM T-Ub, 125 nM E1, 2 μM E2 (UBE2D3 or UBE2N) and 2 μM E3 NEDD4L, or a master solution lacking E3 NEDD4L as a control, was added to sample wells. Samples then received a 3 μL addition of either 250 μM WT Ub, lysine-less Ub, methylated Ub, one of the seven Ub K-only mutants, or one of the seven K-R Ub mutants, resulting in a final concentration of 37.5 μM unlabeled Ub in each sample well. The control lacking NEDD4L received WT Ub. FP was monitored for 5 cycles, before initiating the Ub cascade with a 1 μL addition of ATP to a final concentration of 5 mM. FP was monitored for an additional 75 cycles over the course of approximately 2 h. Each sample was prepared in triplicate, with the FP values averaged at each timepoint. The FP value at each time point was normalized to the average of the sample's initial 5 FP values before ATP addition (0% activity), and to the final 5 FP values of the WT Ub sample (100% activity).

### DUB Treatment

Ub chains were created in a master solution resulting in final concentrations of 25 mM sodium phosphate (pH 7.4), 150 mM sodium chloride, 10 mM MgCl_2_, 100 nM T-Ub, 125 nM E1, 2 μM E2 UBE2D3, 2 μM E3 NEDD4L, 50 μM WT Ub, and 5 mM ATP, or a master solution lacking ATP as a control. The master solutions were incubated at 37°C for 1 h while shaking at 500 rpm, and then distributed into sample wells containing a final concentration of 10 mM DTT. FP signal was monitored for 10 cycles before DUB addition.

DUBs were incubated at room temperature for 15 min in 25 mM sodium phosphate (pH 7.4), 150 mM sodium chloride, 10 mM DTT, and 30 mM EDTA. After incubation, DUBs were added to the sample wells containing NEDD4L-generated Ub/T-Ub ubiquitination products. In this assay AMSH had a final concentration of 250 nM, while ChlaDUB1, OTUB1, OTULIN, and USP21 had final concentrations of 600 nM. Following DUB addition, deubiquitination was monitored for approximately 2 h. The FP values at each timepoint were normalized to the sample's averaged FP value prior to DUB addition (0% activity), and to a corresponding control sample that contained all components except for ATP, representing an unconjugated Ub signal (100% activity).

### Data Analysis

Data was first analyzed using the MARS data analysis software from BMG LABTECH. The fluorescence polarization values were calculated by the MARS software using the equation:

(1) $$\begin{array}{l} FP=1000\times \frac{\|-⊥}{\|+⊥} \end{array}$$

where || and ⊥ are the measured values from the parallel and perpendicular channels, respectively, both in units of mP. Averages and standard deviations of FP data were calculated and plotted using GraphPad Prism.

Z′ values were calculated for each assay according to the equation:

(2) $$\begin{array}{l} Z^{\prime }=1-\;\frac{\left(3\sigma_{c+}+\;3\sigma_{c-}\right)}{\left|u_{c+}\;-\;u_{c-}\right|} \end{array}$$

where μ_c+_ and μ_c−_ are the means of the positive and negative controls, respectively, and σ_c+_ and σ_c−_ are the standard deviations of the positive and negative controls, respectively.

## Results

FP is a sensitive measure of a molecule's tumbling behavior in solution. Though primarily used to study protein-protein interactions, previous studies using FP to discriminate monomeric Ub from polyUb chains (Ye et al., 2011; Keusekotten et al., 2013; von Delbrück et al., 2016; Mot et al., 2018) led us to reason that FP could be used to monitor the passage of fluorescent Ub through the entire ubiquitination cascade (Figure 1A). Using Ub labeled with tetramethylrhodamine (TAMRA) at its amino-terminus (T-Ub), we could show that conjugation onto the E1 active site resulted in a large shift in FP (Figure 1B, step 1). Addition of the E2 Ub-conjugating enzyme UBE2D3 led to rapid formation of the E2~Ub conjugate, with an intermediate molecular weight and corresponding FP value (Figure 1B, step 2). Subsequent addition of the bacterial HECT-type E3 ligase NleL resulted in a modest increase in FP (Figure 1B, step 3), which dramatically increased over time following the addition of excess unlabeled Ub into the system (Figure 1B, step 4). These Ub modifications (most likely polyUb) could then be removed with the nonspecific DUB USP21, which was evident by a decrease in FP value with time (Figure 1B, step 5). Thus, the entire Ub conjugation and deconjugation cycle could be observed in real time simply by tracking the FP of labeled Ub. Our subsequent work with this method focused on analyzing the discrete steps of Ub conjugation and deconjugation to evaluate the utility of UbiReal for measuring activity and specificity.

**Figure 2:**
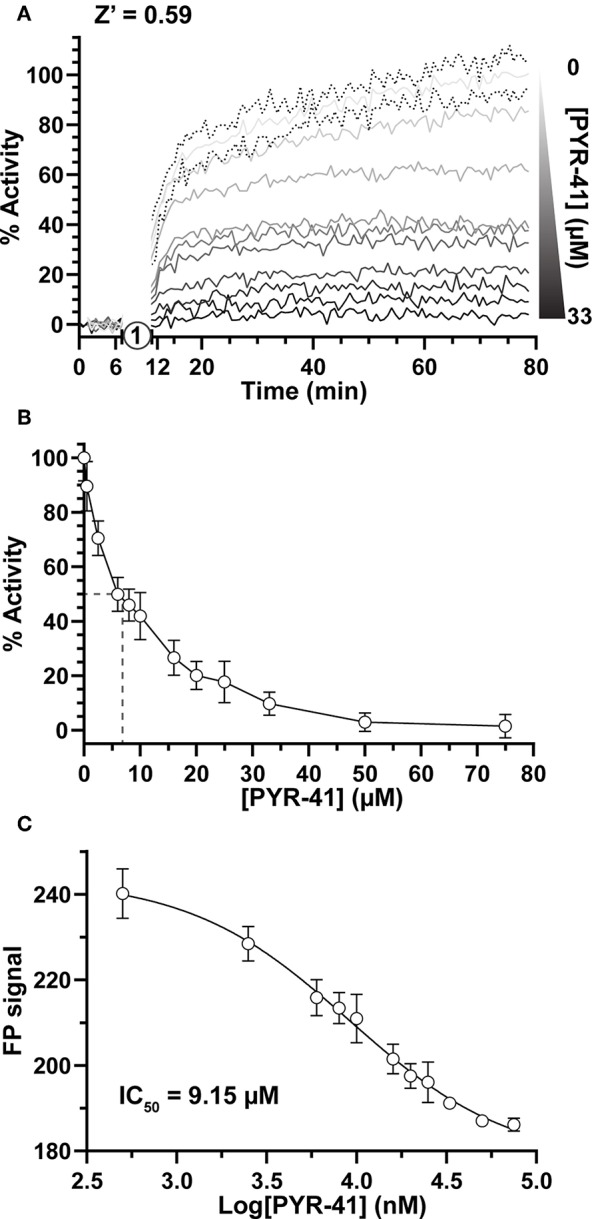
Small molecule inhibition of E1 Ub-activating activity. **(A)** E1~T-Ub complex inhibition by PYR-41 monitored over time with the UbiReal assay. “1” indicates the addition of ATP to initiate formation of the E1~T-Ub complex. Increasing concentrations of PYR-41 correspond to reduced E1~T-Ub complex formation, represented by % Activity. Data points are normalized to FP values after PYR-41 addition, but before ATP addition, and to the FP signal from samples treated with DMSO instead of PYR-41, representing 100% activity. Connected lines represent Mean values, while representative error of ± SD is shown for the 0 μM PYR-41 (DMSO addition) sample. Data are the average of 3 technical replicates for each concentration and representative of all PYR-41 inhibition experiments. **(B)** Inhibition of E1~T-Ub complex by PYR-41 represented using the end-point FP values at each PYR-41 concentration. The dashed lines represent the approximate PYR-41 concentration at which 50% E1~T-Ub complex inhibition occurs. Data are normalized as in **(A)**. Data are from 3 separate experiments that each include 3 technical replicates for all PYR-41 concentrations (see section Methods). Data are reported as Mean ± SD. **(C)** IC_50_ graph generated using the end-point FP values as in **(B)**. Data was fitted and an IC_50_ value was calculated using a non-linear regression in GraphPad Prism. Data are from the same experiments as **(B)**, with data reported as Mean ± SD.

Focusing first on Ub activation, we measured E1 activity in response to increasing concentrations of the previously described chemical inhibitor PYR-41 (Yang et al., 2007). By incubating E1 with PYR-41 and subsequently initiating the reaction with ATP (Figure 2A, step 1), E1~T-Ub complex formation could be monitored over time (Figure 2A). Data were normalized to FP values before ATP addition (0%) and to the endpoint of the DMSO-only control (100%). Effects of PYR-41 addition could be observed as a loss in activity ranging from no to complete inhibition (Figure 2A). We noted a moderate degree of variability in our FP measurements, possibly arising from the addition of DMSO, but still calculated an overall Z' value of 0.59 [a measure of signal-to-noise in HTS where values in the range of 0.5–1.0 are considered “excellent” (Zhang et al., 1999)]. E1 activities reported by our assay showed a logarithmic trend with increasing concentration of PYR-41 (Figure 2B). Using a non-linear regression, an IC_50_ value for inhibition of E1~Ub conjugation by PYR-41 under our assay conditions was determined to be 9.15 μM (Figure 2C), in agreement with previously reported values (Yang et al., 2007).

**Figure 3:**
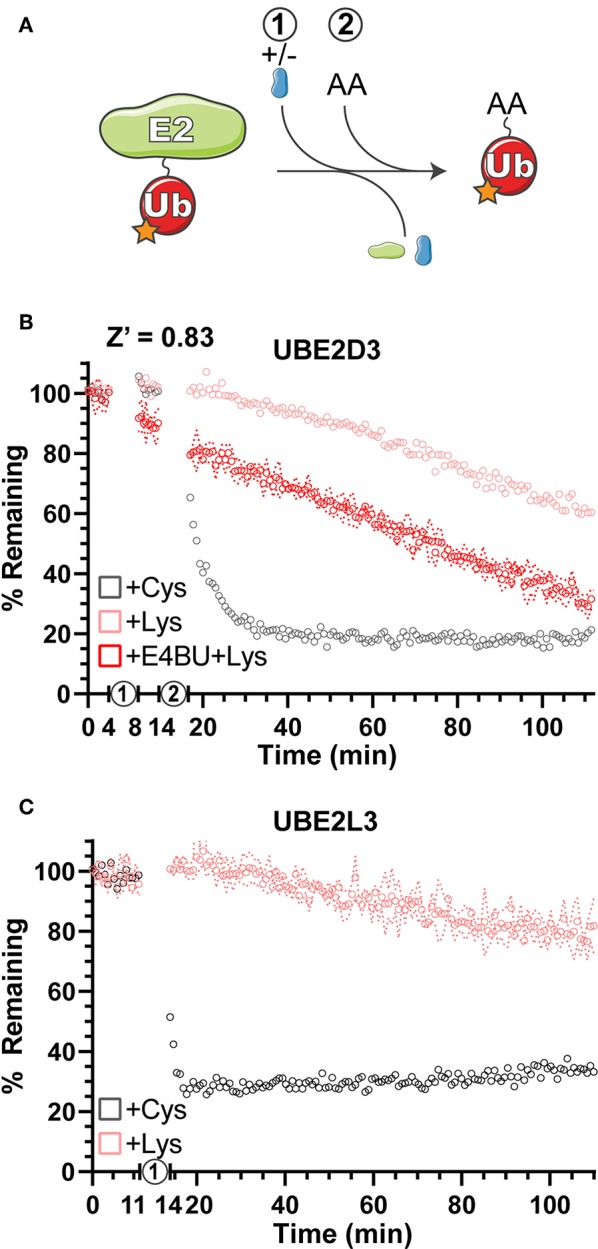
Amino acid reactivity and activation of E2 Ub-conjugating enzymes. **(A)** Reaction schematic depicting E2~F-Ub discharge onto a substrate amino acid (AA). **(B)** Monitoring discharge of the E2 Ub-conjugating enzyme UBE2D3 from E2~F-Ub to Lys~F-Ub or Cys~F-Ub using UbiReal. “1” indicates the addition of ± E4BU, and “2” indicates the addition of amino acid. Samples are monitored over time, and data are normalized to samples not treated with amino acid (100% remaining) and samples with amino acid but lacking ATP (0% remaining). Data are reported as the Mean values from an experiment with 3 technical replicates, and representative error of Mean ± SD is reported for the UBE2D3+E4BU+Lys sample. **(C)** Discharge of UBE2L3~F-Ub to Lys~F-Ub or Cys~F-Ub over time. “1” represents the addition of amino acid. Data are reported and normalized as in **(B)**, with representative error reported for the UBE2L3+Lys sample.

Gel-based Ub discharge assays have been used previously to measure the ability of E2 enzymes to transfer Ub onto free amino acids as a simplified model for substrates (Wenzel et al., 2011; Pruneda et al., 2012; Buetow et al., 2018). Using Ub labeled with fluorescein at all primary amines (F-Ub), amino acid reactivity and specificity were measured for the E2 enzymes UBE2D3 and UBE2L3 (Figure 3A). Using activated E2~F-Ub as a starting material, the free amino acids Cys and Lys were added and discharge was measured as the return to unconjugated F-Ub FP values over time (Figures 3B,C). As expected from previous work (Wenzel et al., 2011), UBE2D3 demonstrated the ability to transfer F-Ub to both Cys and Lys amino acids (Figure 3B), whereas UBE2L3 was largely Cys-specific (Figure 3C), indicating that it cannot directly ubiquitinate substrate Lys residues but must act through a HECT/RBR E3 intermediary. As an E2 that can directly ubiquitinate Lys residues, UBE2D3 functions with RING/U-box E3 ligases to efficiently transfer Ub. Addition of the U-box E3 ligase E4BU to the UBE2D3~F-Ub conjugate already promoted discharge of the thioester linkage (Figure 3B, step 1), and in the presence of free Lys resulted in an enhanced rate of Ub transfer (Figure 3B, step 2) as observed in previous gel-based assays (Pruneda et al., 2012). Overall, the UbiReal method provided a straightforward approach for observing the specificity and activation of E2 Ub-conjugating enzymes.

**Figure 4:**
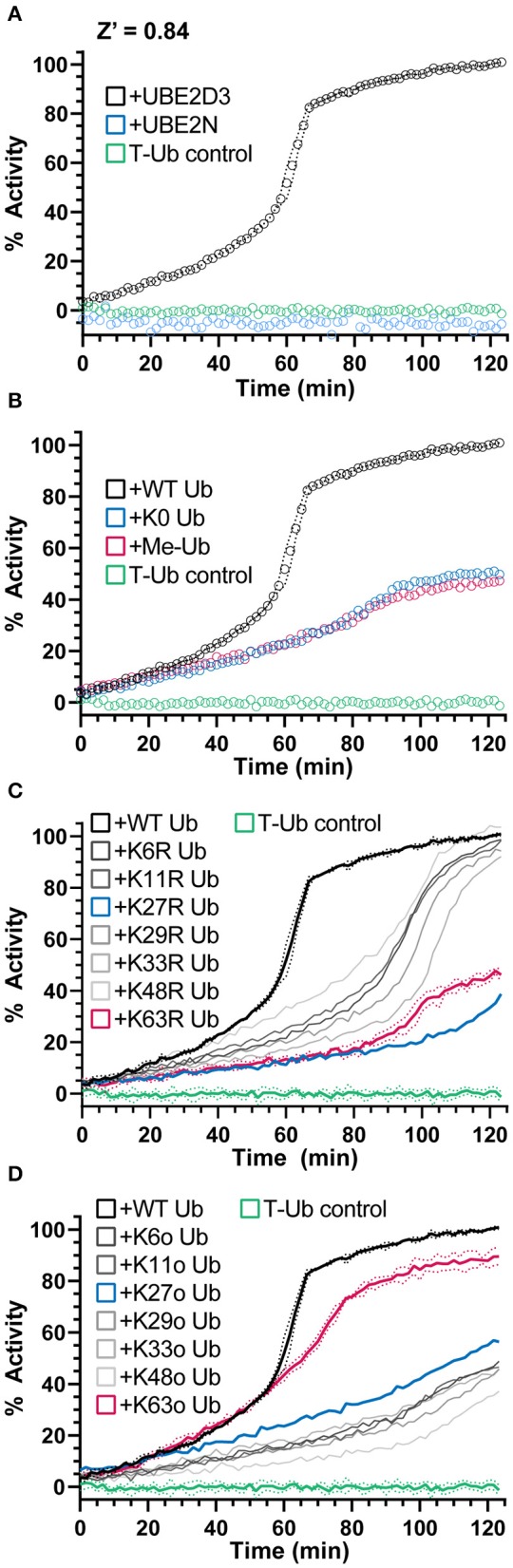
NEDD4L E3 polyUb ligation and chain specificity. **(A)** Ub chain ligation by K63-chain specific NEDD4L and the E2 enzyme UBE2D3 or UBE2N monitored over time using UbiReal. Reactions are initiated with ATP at time 0. Data for each sample is normalized to its starting FP value before ATP addition (0% activity) and to the final values of the NEDD4L+UBE2D3+WT Ub sample (100% activity). Data are reported as the Mean from an experiment with 3 technical replicates, with representative error reported as Mean ± SD for the NEDD4L+UBE2D3+WT Ub sample. Data for this and subsequent panels were collected together, and the UBE2D3 and T-Ub data are included as positive and negative controls, respectively, in the panels to follow. **(B)** Monitoring polyUb vs. monoUb formation by NEDD4L and UBE2D3 over time. Reactions are initiated with ATP at time 0. K0 Ub is a mutant lacking all Lys; Me-Ub is methylated at each primary amine. Data are reported and normalized as in **(A)**. **(C)** Ub chain ligation by NEDD4L and UBE2D3 over time using a mutant KR Ub panel that has individual Lys residues mutated to Arg (K63R has every Lys except K63, etc.). Reactions are initiated with ATP at time 0. Data are reported and normalized as in **(A)**, with representative error reported as Mean ± SD for some samples. **(D)** The same experiment as **(C)**. but using a mutant Ko Ub panel that contain only a single Lys residue, with all other Lys mutated to Arg (K63o contains only K63, etc.). Data are reported and normalized as in **(A)**, with representative error reported as Mean ± SD for some samples.

E3 ligases traditionally facilitate the final transfer of Ub onto a substrate, but even in the absence of substrate, E3s will often autoubiquitinate themselves or form free Ub chains *in vitro*. Gel-based assays typically report this activity as a “smear” of Ub modifications in the high molecular weight range that is difficult to reliably quantify. As shown in Figure 1B, the UbiReal approach can be used to monitor E3 ligase activity, particularly after the addition of excess unlabeled Ub that continually builds high molecular weight products that contain T-Ub. Using a different HECT-type E3 ligase, NEDD4L, we could again show robust ubiquitination activity that builds with time (Figure 4A). Importantly, this activity was dependent upon known E2-E3 specificity (Kamadurai et al., 2009), as UBE2D3 could generate large ubiquitinated products with NEDD4L but not UBE2N, an E2 that typically functions with UBE2V2 and RING/U-box ligases (Figure 4A).

NEDD4L, as a HECT-type E3 ligase, controls the context of the final ubiquitinated product, i.e., mono- vs. polyubiquitination as well as the Ub chain specificity (Kim and Huibregtse, 2009). As the bulk of the ligase-dependent ubiquitination signal develops after an influx of unlabeled Ub, we sought this opportunity to instead supplement mutated Ub that could inform on the type of Ub modification. By supplementing the reaction with K0 Ub (in which all seven Lys residues are mutated to Arg) or Me-Ub (in which all primary amines have been methylated), the FP signal rose to only 50% of that observed with WT Ub (Figure 4B). Interestingly, this result suggested that approximately half of the FP signal originated from mono- or multi-mono-autoubiquitination, with the remaining activity originating from chain-building activity of NEDD4L.

To probe the type of polyUb chain formation observed in the NEDD4L reaction, two additional sets of mutated Ub were used. The first set consists of all possible Lys-to-Arg mutants, each eliminating one potential site of chain linkage (e.g., K63R). As expected for the K63-specific ligase NEDD4L, addition of the K63R mutant Ub decreased the ubiquitination signal to levels consistent with the K0 Ub control, whereas most other Lys-to-Arg mutants had little effect on product formation (Figures 4B,C). Interestingly, the K27R mutant Ub also produced less ubiquitination signal and could indicate a local disruption in the Ub structure (K27 is the most buried of all Lys) or in some interaction with the conjugation machinery. The second set consists of Ub K-only mutants, in which six of the seven Lys residues have been mutated to Arg leaving only one behind (e.g., K63o). With this panel, only the K63o mutant could generate a ubiquitination signal similar to WT, whereas all other mutants behaved like the K0 Ub control (Figures 4B,D). Together, these experiments confirm the K63 specificity of NEDD4L (Maspero et al., 2013) and illustrate the utility of the UbiReal approach for studying E3 ligase activity.

**Figure 5:**
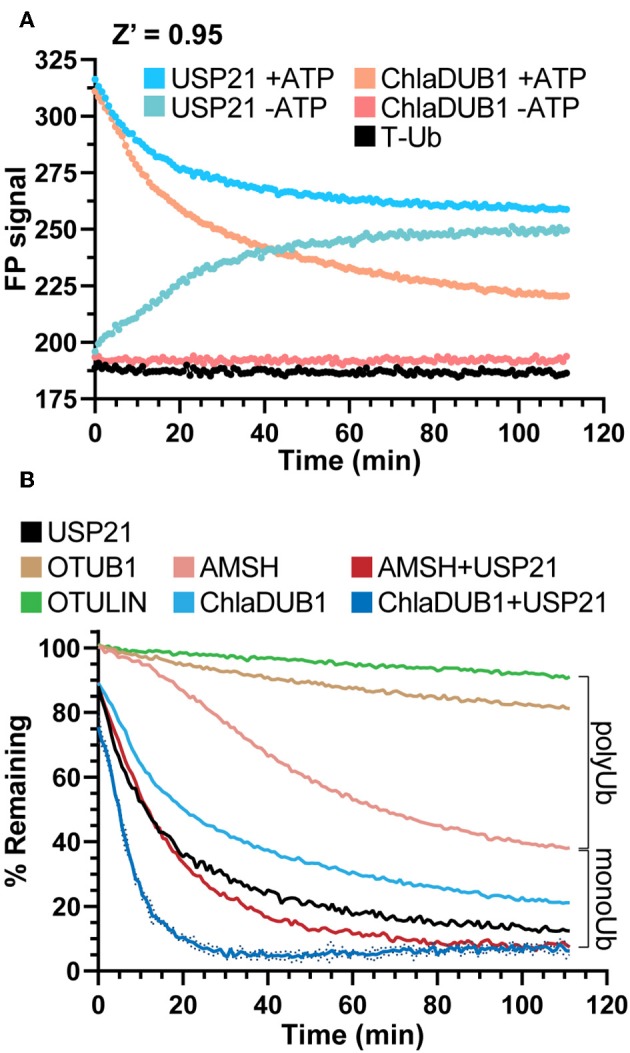
DUB activity and UbiCRest analysis of polyUb chain types. **(A)** Monitoring deubiquitination over time with DUBs USP21 and ChlaDUB1 using UbiReal. Curves show the raw FP signal of USP21 or ChlaDUB1 activity against samples containing a NEDD4L ligation mixture ± ATP. +ATP samples represent DUB activity against NEDD4L-generated chains while –ATP samples represent background FP signal where no ligation activity could occur. Reactions were initiated by addition of the DUB at time 0. +ATP samples are reported as the Mean of 3 technical replicates while –ATP samples are a representative single sample. **(B)** Monitoring deubiquitination over time with DUBs USP21, OTUB1, OTULIN, ChlaDUB1 ± USP21, and AMSH ± USP21. The polyUb and monoUb brackets indicate the observed contributions of monoUb and polyUb in the NEDD4L ligation mixture. Data are from the same assay as **(A)**. and are reported in the same manner, with representative error reported as Mean ± SD for the ChlaDUB1+USP21 sample.

In our initial experiments addressing the measurement of DUB activity, we observed an incomplete reduction in FP signal using the DUB USP21 (Figure 1B, step 5), though we expected the non-specific activity of USP21 toward both mono- and polyubiquitination (Hospenthal et al., 2015) to return the FP signal to unconjugated T-Ub values (Figure 1A) To understand the discrepancy, several control experiments were prepared to observe the behavior of USP21 under our assay conditions. The Ub conjugation assay components (T-Ub, E1, UBE2D3, NEDD4L, and WT Ub) were incubated with or without ATP, and this was used as the starting substrate to which each DUB was added. Interestingly, when combined with the –ATP sample that could not support Ub conjugation, the USP21-treated sample increased in FP over time, most likely a result of noncovalent interactions between T-Ub and USP21 (Figure 5A). The +ATP sample treated with USP21 decreased to the same FP value as the –ATP sample by the end of the time course, indicating that complete deubiquitination had occurred (Figure 5A). For other DUBs like ChlaDUB1, an effector protein from *Chlamydia trachomatis* that preferentially cleaves K63 chains (Pruneda et al., 2016), the background present in the –ATP samples was not as significant as for USP21 (Figure 5A), but a –ATP sample was prepared nonetheless for each DUB in subsequent experiments to control for potential background binding. These experiments established a key foundation for the following DUB assays, but also suggest that USP21 most likely suffers from product inhibition resulting from a high affinity for free Ub, as has previously been shown for USP2 (Renatus et al., 2006).

UbiCRest is a powerful method that has been used to determine the type of ubiquitination present in a sample through treatment with Ub chain-specific DUBs (Hospenthal et al., 2015). Though normally interpreted using a gel-based readout, we applied the UbiCRest strategy to NEDD4L-generated ubiquitination in order to detect DUB activity through the release of T-Ub. Using AMSH, an endosome-associated DUB that preferentially cleaves K63 chains, cleavage of the NEDD4L assembly was observed to approximately 40% remaining FP signal, and when combined with USP21, complete cleavage was observed (Figure 5B). This result suggested that while AMSH can cleave the K63-linked polyUb, it cannot remove monoUb modifications which likely account for the ~40% remaining signal (consistent with Figure 4B). ChlaDUB1 alone cleaved the Ub assembly to around 20% remaining, and together with USP21 could completely remove all modifications (Figure 5B). This suggested that ChlaDUB1, unlike AMSH, appears to be more promiscuous toward monoubiquitination. K48-specific OTUB1 and Met1-specific OTULIN were used as negative controls that should not have deubiquitinating activity toward NEDD4L-generated chains, and the slight drift observed in these samples could be an experimental artifact or low-level cleavage (Figure 5B). Taken altogether, the UbiCRest approach for characterizing ubiquitination in our assay was effective at identifying both the amount and type of polyUb present.

## Discussion

In an effort to address a longstanding need for a robust HTS for Ub conjugation, we have designed and tested UbiReal as a real-time assay for monitoring all ubiquitination activities. UbiReal uses commercially-available fluorescently-labeled Ub to track the progression through E1, E2, and E3 enzymes by the molecular weight and resulting fluorescence polarization changes associated with each step. Using this approach, ubiquitination activities can be observed in a highly parallel manner that consumes remarkably little material (on the order of 10 ng of labeled Ub per reaction). Unlike other more specialized approaches, UbiReal offers a universal method that allows the user to directly observe each consecutive step of Ub conjugation, from the E1 through to the E2, E3, and substrate ubiquitination. Furthermore, the ubiquitination products assembled using this method provide a more complex, realistic substrate that can be used to monitor DUB activity. In our trials, we found that UbiReal was able to provide both quantitative measurements of activity as well as qualitative insights into mechanisms and specificities of Ub transfer.

As a test of its power to assay small molecule modulators, we used UbiReal to monitor the inhibition of E1 Ub-activating function in response to the PYR-41 inhibitor. In a dose-response experiment, we determined the IC_50_ of PYR-41 to be 9.15 μM, consistent with the reported estimation of <10 μM from a radioactive gel-based assay (Yang et al., 2007) and a fluorescent activity-based probe assay (An and Statsyuk, 2013). We chose to analyze this experiment as an endpoint assay as PYR-41 is an irreversible inhibitor, but the same experiment provides kinetic information as well and could easily be used to measure effects of competitive inhibitors on initial velocity. From our experimental control data, we determined Z' values in the range of 0.59–0.95 for all of our directed UbiReal experiments measuring E1, E2, E3, and DUB activities, indicating that under these conditions UbiReal provides excellent signal-to-noise ratios that are compatible with HTS. With minor adjustments, we expect that the UbiReal approach could be an effective HTS for any regulator of ubiquitination.

The UbiReal method was also useful for determining several qualitative aspects of Ub conjugation and deconjugation. Simplified amino acid reactivity assays provide a straightforward measure of E2 enzyme activity, and we showed that UbiReal is able to recapitulate both the reactivity profiles of several E2 enzymes as well as the reactivity enhancement mediated by RING/U-box E3 ligases. By supplementing the reaction with unlabeled Ub, we observed robust E3 ligase activity in the form of autoubiquitination. By changing the nature of the supplemented Ub, we were able to distinguish mono- vs. polyubiquitination as well as determine the preferred Ub chain type. To corroborate this chain type determination, we applied a simplified UbiCRest approach to our assay in order to observe which chain-specific DUBs could reduce the FP of our samples back to a monoUb value. Just as in the gel-based UbiCRest approach, by treating with DUBs singly or in combination, we observed complete, partial, or negligible collapse of FP values that indicate both the chain type and mono- vs. polyUb architecture present in our complex ubiquitinated sample. These proof-of-principle studies indicate the applicability of UbiReal across the entire Ub cascade. Though we focused on aspects of Ub transfer specificity, the same approach could be used to study the mechanisms of Ub transfer, for example by incorporating structure-guided mutations. As an alternative to conventional gel-based assays, UbiReal can provide quantitative information in less time with less material. Furthermore, by separating each stage of Ub transfer, in one assay the user can isolate the precise step (e.g., E2~Ub formation vs. discharge) that is affected by perturbations such as mutations or small molecule modulators.

Existing HTS for Ub conjugation have primarily focused on observation of the final ubiquitinated substrate. The bulk of these methods rely on either direct detection of Ub following enrichment of substrate (e.g., ELISA), or detection of Ub in close proximity to substrate (e.g., FRET or AlphaScreen). Because these assays are specialized for detecting ubiquitinated substrate, they are not well-suited for monitoring each stage of Ub conjugation separately. Fluorescence polarization provides the unique opportunity to track Ub based on its tumbling rate in solution vis-à-vis its molecular weight. This approach has been used to track different aspects of the Ub system before. By either placing the label on the substrate or the Ub itself, E2- or E3-mediated polyUb chain formation has been observed by increasing FP (von Delbrück et al., 2016; Mot et al., 2018). Specialized Ub substrates can also be used to directly monitor the activities of HECT- or RBR-family E3 ligases by FP, in the absence of E1 or E2 enzymes (Krist et al., 2016; Park et al., 2017). DUB activities have been measured using defined, fluorescently-labeled Ub chains (Ye et al., 2011; Keusekotten et al., 2013). Interestingly, FP has even been used to track the proteasomal degradation of ubiquitinated substrates (Bhattacharyya et al., 2016). It is based on these observations that we developed UbiReal as a generalized approach to observe all consecutive steps of both Ub conjugation and deconjugation in real time.

As with any method, UbiReal does have certain caveats. The most glaring is the dependence on large differences in molecular weight that are required for significant changes in FP. In particular, size similarities between E2, E3, or substrate proteins could pose challenges. One solution to this problem could be to incorporate protein tags, such as GST, to shift molecular weights. A second caveat to our approach is the location of the fluorophore. Though labeling the amino-terminus is routine practice and practically inert for most purposes, it obviously precludes the formation of Met1-linked polyUb. In this case, we expect that the label could instead be conjugated through maleimide chemistry to a Cys residue introduced at, for example, position 20 (von Delbrück et al., 2016). Our tests with two varieties of fluorescent Ub (F-Ub and T-Ub) suggest that other dyes and sites of attachment will also be amenable to UbiReal. Lastly, we recognize that our ability to track fluorescent Ub through each stage of the conjugation process requires a molar excess of conjugating enzymes, which may preclude certain applications of the method. However, if the desired readout does not depend on observing each transfer event (e.g., E2~Ub formation vs. polyUb formation), the concentrations of each enzyme component can be tuned to suit the reaction requirements.

In sum, we present a simple method that addresses a need for a universal HTS for Ub conjugating activity. UbiReal requires no specialized reagents, only a fluorescently-labeled Ub which is readily available in multiple forms. With only minor optimization, we were able to apply the UbiReal method to measure E1, E2, E3, and DUB activities in separate, controlled experiments. We believe that the robust and scalable nature of this assay will make it useful in HTS for small molecule modulators, and its convenience and quantitative nature makes it a compelling alternative to the conventional gel-based assays for mechanistic work.

## Data Availability Statement

The datasets generated for this study are available on request to the corresponding author.

## Author Contributions

TF and JP conceptualized the approach, analyzed the data, and wrote the manuscript. TF performed all experiments.

### Conflict of Interest

The authors declare that the research was conducted in the absence of any commercial or financial relationships that could be construed as a potential conflict of interest.

## Abbreviations

Ub: ubiquitin
FP: fluorescence polarization
DUB: deubiquitinase
HTS: high-throughput screen
T-Ub: TAMRA-Ub
F-Ub: Fluorescein-Ub.
